# Assessing How Fact‐Checks Influence Accuracy and Consensus Judgments: Evidence From the Olympics

**DOI:** 10.1111/risa.70293

**Published:** 2026-06-25

**Authors:** Morgan Wack, Jinan Allan, Brandon Boatwright, Gregory Cranmer

**Affiliations:** ^1^ Department of Communication and Media Research (IKMZ) University of Zurich, Zurich, Switzerland; ^2^ Department of Psychology Clemson University, Clemson, South Carolina, USA; ^3^ Department of Communication Clemson University, Clemson, South Carolina, USA

**Keywords:** consensus effects, content moderation, fact‐checking, misinformation, sports

## Abstract

Fact‐checks have become a popular intervention aimed at tackling misinformation, yet concern has persisted related to the potential for fact‐checking to inadvertently amplify false narratives—a phenomenon commonly referred to as a “backfire effect.” At the same time, recent evidence from research on repeated claims has suggested that there is a risk that fact‐checks may also generate misperceptions regarding societal beliefs—or an “illusory consensus” effect. This paper presents findings from an ecologically valid two‐wave experimental study that tested whether repeated fact‐checking interventions could cause either of these negative outcomes. To further enhance the validity of the design, we draw on quota‐balanced samples of participants from the United States, France, and South Africa (*N* = 680), who were presented with a set of true and false posts taken from online discussions regarding the 2024 Summer Olympic Games in Paris, France. Outcome measures focused on personal accuracy judgments (i.e., how accurate participants deemed each claim) and consensus estimates (i.e., whether their fellow citizens would believe each claim). After the Olympics concluded, participants were asked to assess both new and previously reviewed statements. Consistent with prior work, results indicate no evidence of the backfire effect (d = 0.048). The findings also provide evidence of a small “illusory consensus” effect (d = 0.125), whereby participants who had previously seen fact‐checks were more likely to estimate that the wider population would accept these debunked claims as accurate. Supplementary analyses of true statements reveal that fact‐checks produced large, durable improvements in accuracy judgments (d = 0.71–0.91), suggesting that fact‐checking was substantially more effective at reinforcing correct beliefs than it was at generating unintended consequences. Item‐level analyses further reveal heterogeneity across misinformation narratives, indicating that the specific content of false claims may moderate both the persistence of corrections and consensus distortions. Implications for fact‐checking strategies and public perceptions of misinformation, including the need for further studies which incorporate group dynamics, are discussed and debated.

## 1 Introduction

High‐profile events, like elections and the Olympic Games, offer fertile ground for the rapid diffusion of falsehoods as a result of the attention they attract and the uncertainty in processes and outcomes they generate (Starbird et al. 2023; Wack et al. 2025). In response, fact‐checking has emerged as an important corrective tool, with organizations and independent verifiers striving to dispel false claims before they can exert lasting effects on public perceptions. Yet, as fact‐checking interventions have gained momentum as the primary method for debunking falsehoods online (Lazer et al. 2018) questions remain regarding their capacity to mitigate misinformation without generating support for specific false narratives.

Research on fact‐checking and misinformation has highlighted the potential of corrective information to reduce the acceptance of false claims, but the findings are mixed. Several studies demonstrate that providing factual corrections can lead to improvements in belief accuracy (Ecker et al. 2019). Others, however, raise the possibility of a “backfire effect,” wherein individuals become more entrenched in their erroneous beliefs following exposure to a correction (Nyhan and Reifler 2010; Pluviano et al. 2017). Although many of these backfire scenarios have failed to replicate in later work (Ecker et al. 2023; Prike et al. 2023), an important and underexamined consideration is how fact‐checking interventions persist, or fail to persist, when they are delivered across multiple time points in ecologically valid settings.

Moreover, new to this paper, we examine for the first time in the context of fact‐checking, the case in which repeated information alters meta‐perceptions, or beliefs regarding how widely false claims are accepted by others. While direct fact‐checks may influence personal perceptions, the repeated nature of correction messaging may additionally lead to the perception that these rumors are more common in wider society. As such, the current work extends the field of fact‐checking research on the influence of consensus perceptions. Consensus perceptions have been linked directly to behavioral outcomes (Ross et al. 1977; Marks and Miller 1987; Prentice and Miller 1993; R. Cialdini and Trost 1998), including for climate change adaptation actions (Goldberg et al. 2020; van Valkengoed et al. 2022; Andre et al. 2024), vaccine acceptance and health‐related behavior (Batteux et al. 2023; R. B. Cialdini et al. 1991; Prentice and Miller 1996), and labor force participation decisions (Bursztyn et al. 2020). Recent laboratory research has further demonstrated that the mere repetition of information, even without source cues, can increase perceptions that others believe or know that information (Jalbert and Pillai 2024), a finding the authors describe as an “illusory consensus effect,” which has ties to the broader false consensus effect literature (see Ross et al. 1977). The present study extends this finding to the ecologically valid context of fact‐checking during a real‐world contentious event. Specifically, we examine whether repeated exposure to fact‐checks of Olympic‐related misinformation narratives similarly distorts meta‐perceptions of societal beliefs, with implications for how fact‐checking interventions should be designed and deployed.

In extending prior work on the backfire effect and, for the first time, assessing the influence of fact‐checking on consensus outcomes, this study examines how fact‐checks influence both individual accuracy estimates and social meta‐perceptions. To answer these questions, we present a two‐wave experiment that systematically varied the presentation of misinformation about the 2024 Paris Olympic Games. Across three distinct country cohorts and two phases, we test whether (a) fact‐checks produce enduring improvements in belief accuracy over time for originally presented misinformation, and (b) whether fact‐checks influence broader consensus perceptions. This design allows us to explore not only the extent to which fact‐checks shift personal beliefs, but also whether these shifts carry over into beliefs about others' acceptance of misinformation, potentially illuminating mechanisms that might exacerbate or mitigate backfire and illusory consensus effects.

Finally, we expand our analyses by examining individual differences that may influence the uptake of corrections or susceptibility to misinformation. Variables such as digital and AI literacy (Helsper et al. 2020; Wang et al. 2025), statistical numeracy (Cokely et al. 2012), scientific literacy (Rosenthal 2020), and cognitive reflection (Frederick 2005) may moderate participants' reliance on fact‐checks. Including participants from across the United States, France, and South Africa further allows us to examine cross‐national patterns in how these individual difference factors interact with fact‐checking interventions. In addition, we conduct supplementary analyses examining the effects of fact‐checking on true statements. While a growing literature has examined the durability of corrections to misinformation (Guess et al. 2020; Swire‐Thompson et al. 2020), the persistence of fact‐checking effects on verified information has received comparatively little attention. Pennycook et al. (2020) demonstrated that “verified” labels can counteract the implied truth effect within a single session, but whether such verification effects persist across weeks in a naturalistic context remains an open question. Our two‐wave design allows us to address this gap. By investigating both immediate and longer term effects of fact‐checks, as well as exploring participants' meta‐judgments about others' beliefs, our study aims to offer a nuanced view of how misinformation interventions function in an environment where the credibility of the source, the relevance of national identity, and individual cognitive and literacy factors may all shape outcomes.

The following sections outline our experimental design, detailing how misinformation and fact‐checks will be presented, as well as the range of measures used to assess beliefs and consensus estimates. We then describe our hypotheses, which focus on detecting the immediate effects of fact‐checks. By identifying the conditions under which fact‐checks effectively reduce misinformation acceptance, as well as those in which they fail to reduce acceptance, we contribute actionable insights for designing and deploying more effective, context‐sensitive fact‐checking interventions.

## 2 Background and Theory

### 2.1 Fact‐Checking the “Backfire Effect”

The “backfire effect” refers to a hypothesized phenomenon in which individuals, upon receiving corrective information, become more entrenched in their original misconceptions rather than updating their beliefs. This effect has been a focal point in the literature surrounding fact‐checking, particularly in the context of political misinformation and public health narratives. Early studies suggested that corrections could sometimes reinforce false beliefs, particularly among individuals with strong preexisting biases (Nyhan and Reifler 2010).

Recent research has sought to clarify the conditions under which the backfire effect can occur. Despite its prominence, in their initial study, Nyhan and Reifler (2010) documented instances where corrections increased misperceptions among ideologically aligned groups, suggesting that the backfire effect may be more pronounced in politically charged contexts. This result aligns with findings of Reinero et al. (2023), who notes that fact‐checks issued by political out‐group members were more likely to backfire, indicating that the source of the correction plays a crucial role in its effectiveness. At the same time, several studies have shown that the backfire effect is not universally applicable (Swire‐Thompson et al. 2020), with prior observations linked to insufficient reliability across utilized measures (Swire‐Thompson et al. 2022). Consistent with this view, recent investigations have often failed to replicate earlier findings, suggesting that corrections often succeed in reducing misinformation rather than exacerbating it (Prike et al. 2023; Hameleers 2019; Porter and Wood 2021). Ecker et al. (2019), for example, find that while corrections generally enhance belief accuracy, they do not typically lead to familiarity backfire effects, which suggests that the mere act of correcting misinformation does not inherently provoke resistance.

Despite this recent push‐back, the backfire effect has been commonly cited as a reason for shifting away from fact‐checking. While decontextualized evidence from lab and survey experiments have provided evidence against this concern, important boundary conditions remain unexplored regarding the prominence of the backfire effect and related forms of counter‐productive consequences which necessitate the conduct of ecologically valid experiments. In summary, while the backfire effect remains a significant concern in the literature on misinformation and fact‐checking, recent studies indicate that its prevalence may be overstated. At the same time, this work has rarely tested for a backfire effect in an ecologically valid setting, such as the Olympics.

Given that prior research suggests the backfire effect, if it occurs, may be more likely in contexts involving high emotional salience, strong identity relevance, and real‐world stakes (Nyhan and Reifler 2010; Reinero et al. 2023), the Olympic Games provide a particularly strong test case. The misinformation narratives examined in this study span topics including claims about transgender athlete participation, geopolitical security threats, and health‐related mandates that implicate the identity‐based and ideological motivations most commonly associated with resistance to correction. Moreover, the two‐wave design, separated by the focal event, introduces temporal delays and real‐world distractions that more closely approximate conditions under which memory‐based backfire effects have been theorized to emerge. We therefore use this context to provide a rigorous test of whether the backfire effect manifests under ecologically valid conditions.

Hypothesis 1 Individuals will be more likely to believe misinformation that they have previously seen in the context of a fact‐check (i.e., the “backfire” effect).

To add to the existing literature, we predict that we may find a small backfire effect following the debunking of specific misinformation narratives regarding the 2024 Paris Olympic Games given that the research has suggested that certain contexts may be more conducive than others to the induction of the effect. Specifically, the Olympic context involves misinformation narratives with strong identity relevance and partisan dimensions. While the preponderance of recent null findings tempers expectations regarding the magnitude of any backfire effect, this design provides conditions that, if the effect exists, should be conducive to its detection. Evidence against will add to the growing consensus that the backfire effect is an extremely rare phenomenon which does not threaten to undermine the work of active fact‐checkers.

## 3 Consensus Perceptions

Consensus perceptions, also described as third‐party beliefs or meta‐perceptions, refer to second‐order beliefs—that is, what people think other people think. While individuals in democracies often hold accurate opinions about the beliefs of others, distortions can be incited by partisan media reporting (Rathje et al. 2021; Levendusky and Malhotra 2016; Peterson and Kagalwala 2021) and misinformation narratives (Bogart and Lees 2023). Across contexts, inaccurate consensus perceptions can lead to a range of deleterious consequences, including a tendency to self‐censor (Lees and Cikara 2020), the perpetuation of social divides driven by unwillingness to engage with perceived “others” (Lees and Cikara 2020), and support for political violence (Mernyk et al. 2022).

Research into the causes and consequences of misaligned consensus perceptions has emphasized the prominence of localized social networks and communications channels. Vlasceanu and Coman (2022) highlight how social norms significantly influence health‐related belief updates, suggesting that individuals often rely on the perceived beliefs of others when forming their own opinions. This aligns with the findings of Kobayashi (2018), who demonstrated that perceptions of scientific and social consensus independently affect scientific beliefs. In their study, participants' estimates of consensus were shown to predict their beliefs about scientific issues, indicating that individuals often adjust their beliefs based on their perceptions of what others think, rather than basing their opinions solely on factual information.

The dynamics of social influence are further elucidated by Coman et al. (2016), who discuss the concept of mnemonic convergence in social networks. They argue that collective memory and conversation can shape individual cognition, leading to emergent properties of belief systems at the group level. This suggests that when individuals perceive a consensus around a particular belief, they may be less likely to challenge that belief, even if they personally hold doubts. This phenomenon has been identified as a key component of behavior change in real‐world settings, with evidence suggesting that interventions can effectively shift participant behavior even on contentious issues without shifting attitudes when individuals perceive their own beliefs to be unrepresentative of their broader community (e.g., for an example related to inter‐ethnic marriages, see Paluck (2009)). More recently, evidence from Jalbert and Pillai (2024) provides further support that repeated claims not only influence personal beliefs, but also consensus beliefs, regardless of the veracity of the underlying content.

The importance of mis‐ and disinformation to the development of consensus perceptions has been further explored in the literature on Timur Kuran's concept of preference falsification (Kuran 1987, 1998). By focusing on the behavior of individuals in repressive states, research on preference falsification has indicated the power of consensus perceptions (and projections from the state). In extending the concept to related work on pluralistic ignorance, research has detailed how individuals may publicly conform to perceived social norms while privately holding different beliefs, even when their private beliefs are more accurate representations of societal attitudes (Prentice and Miller 1993, 1996). These pressures are particularly salient in politically charged environments where individuals may feel pressured to align with the dominant narratives, thereby distorting their meta‐judgments about others' beliefs (Kuran 1998).

The implications of social endorsement in belief formation are also significant. In their meta‐analysis, Walter and Tukachinsky (2020) revealed how misinformation continues to influence beliefs even after corrections, suggesting that the social context in which information is received can lead to a misperception of consensus. Butler et al. (2023) similarly found that social endorsements can influence the continued belief in corrected misinformation, indicating that individuals may rely on social cues rather than factual corrections when forming their beliefs. In contexts where there is heavy reliance on social validation, fact‐checking process may be at greater risk of igniting a “illusory consensus” effect where the repetition of the claim signals a stronger consensus around specific misinformation narratives due to the emphasis on endorsements within citizen social networks.

In summary, the formation of opinions about opinions involves a complex interplay of social influence, perceived norms, and individual belief dynamics. Where social validation is highly prized, or necessitated due to risk of sanctions, individuals often rely on their perceptions of others' beliefs to inform their own behavior. As a result, even where individual backfire effects don't exist or are minimal, the potential distortion of consensus perceptions caused by fact‐checking efforts could hold similarly impactful real‐world behavioral consequences.

Hypothesis 2 Individuals will be more likely to believe that others believe misinformation they have previously seen in the context of a fact‐check (i.e., the “illusory consensus” effect).

With respect to the illusory consensus effect, we are more agnostic as to the potential for fact‐checks to alter opinions related to societal perceptions. As discussed in the literature on societal perceptions, we expect that the effect of debunking may not extend to perceptions of societal support due in part to the implicit assumption that information only needs to be debunked when it could or has been taken to be true by a broader audience. While fact‐checks may enable individuals to update their own priors, there is no expectation that the wider public is also aware of the provided corrective information. As a result, rather than worry about the backfire effect, we hypothesize that potential distortions to social perceptions poses a larger threat to fact‐checking efficacy.

## 4 Case Selection and Generalizability

To examine whether fact‐checking processes threaten to exacerbate the consequences of misinformation in real‐world contexts we draw on Paris' hosting of the 2024 Summer Olympics and the surrounding global media attention. The selection of the Olympics as the target event for the conduct of the study held several advantages for the ecological validity and generalizability of the study. As noted, the Olympics provide a focal event which draws attention and generates outcome uncertainty. Both factors have been discussed as key vectors for attracting and amplifying misinformation (Kapferer 2011; Prochaska et al. 2023) and have been associated with related high‐profile events, including elections (Starbird et al. 2023; Wack et al. 2025). As a result, the Olympics enabled the study to focus on a high‐profile event while allowing misinformation to be studied in a context which, while directly applicable to interest in broader social‐political forms of mis‐ and disinformation, held a far lower likelihood of inducing negative impacts among participants.

Beyond serving as a stand‐in for more contentious high‐engagement events, the Olympics also provided the study with sufficient global attention to identify a diverse selection of real‐world misinformation narratives related to a single event. To our knowledge, all prior survey studies on the backfire effect have studied its potential impact on decontextualized misinformation which often take the form of individual falsehoods. Moreover, the vast majority of these studies are conducted as one‐off experiments. While these studies are often designed to include an intra‐survey “cooling” period to separate initial debunking from secondary exposure, these designs do not sufficiently model the randomness of the real‐world environments in which individuals are most likely to encounter false information. While our experimental design remains confined to a survey experimental design, we believe its two‐wave structure, which is dissected by the action of the Olympic Games, more closely approximates the distractions and repeat‐encounter dynamics found in conceptions of the backfire effect literature.

To further align the design of the study with the worries of critics and concerned fact‐checking researchers, we opted to include a range of both factual and false statements of varying emotional and partisan intensity as our stimuli. As discussed in more detail in the next section, by presenting participants from several attending countries with a diverse set of stimuli we aimed to better approximate the variability in the veracity of information available to media consumers across the diverse online media environments characteristic of the internet age (Burkhardt 2017). In all, while no substitute for data from natural experiments, the emphasis on the Olympic Games enabled us to design, by comparison, a more ecologically valid experiment to study the potential negative consequences of fact‐checking during a highly applicable global event.

## 5 Research Design and Data

### 5.1 Timeline and Summary Statistics

Prior to assessing our hypotheses related to the backfire and consensus effects in an ecologically valid setting, we pre‐registered our research design.^1^ All design details and analyses follow the preregistration unless stated otherwise.

**FIGURE 1:**
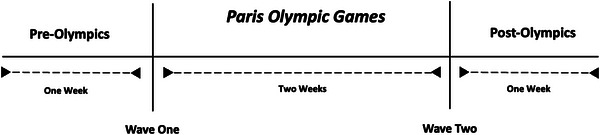
Project timeline.

To test our hypotheses, we recruited a sample of 1500 participants using Prolific from across the three countries competing in the 2024 Olympic Games, with 500 targeted from each of South Africa, the United States, and hosts France. These surveys, which were approved by Clemson University's Office of Research Compliance (Study ID: IRB‐00000481), were identical other than the translation of the survey into French for participants based in France. As illustrated in Figure 1, the first wave of the survey commenced on July 24, 1 week prior to the Olympic's opening ceremony (July 26). Following the Olympics, the second wave was conducted on August 15, 1 week after the Olympic's closing ceremony (August 11).^2^ Across the three groups, participants had an average completion time across surveys of 1090 s (18 min).^3^

Following data collection, we eliminated participants who failed to complete both waves of the survey and participants who failed an included attention check. In all, 606 people were included in the final analyses for the backfire and illusory consensus analyses. Of these participants, 228 were from South Africa, 191 were from France, and 187 were from the United States. These numbers represent the persistence of participants from target samples of 250 for each country. While 500 were initially targeted, a second set of surveys was conducted on misinformation narratives related to the main topics to determine whether there might be “spillover” to related domains for each effect (250 for each condition by country). We found no significant effects in either of these supplementary conditions, which have been reported in the Appendix in the Supporting Information.

### 5.2 Study Design

**FIGURE 2:**
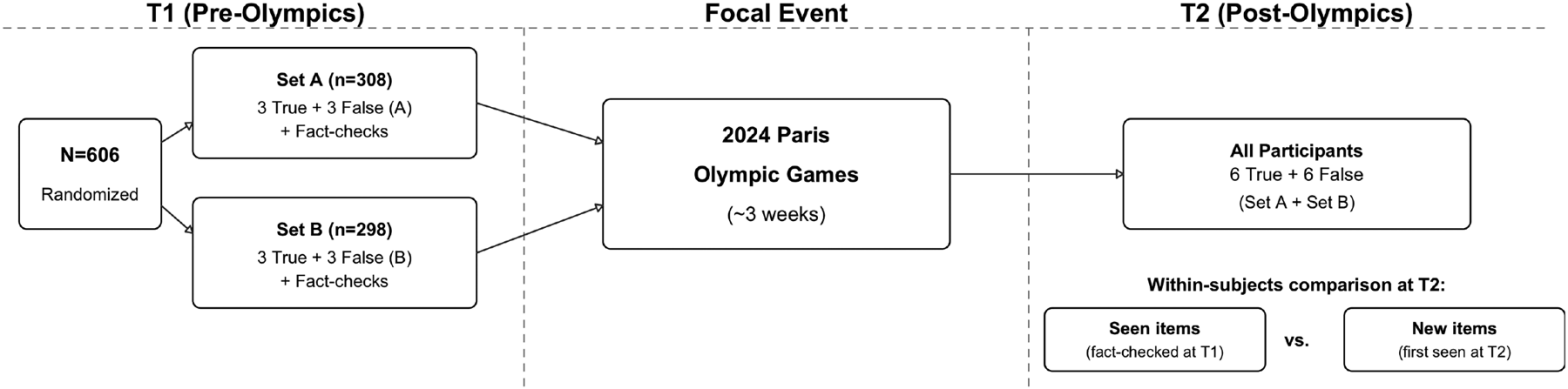
Experimental design overview.

Participants were randomly assigned to see one of two sets of three false claims (Set A or Set B) alongside three true filler statements at $T_{1}$. At $T_{2}$, all participants evaluated all six false claims plus six true statements (see Table 1). The primary comparison is within‐subjects. For each participant at $T_{2}$, we compare responses to previously seen (fact‐checked) items versus new items. While the assignment to Set A versus Set B constitutes a between‐subjects factor, our primary analytical tests exploit the within‐subjects contrast at $T_{2}$, where each participant serves as their own control. Figure 2 provides a visual overview of this two‐wave structure.

To ensure balance across groups, we split false statements between groups by topic. The groups comprised a diverse set of statements and were split to limit thematic overlap between statements in any one group. Set A contained claims related to a CIA metro warning, Esports inclusion, and transgender athlete withdrawal; Set B contained claims about Celsius energy drinks, COVID‐19 vaccine requirements, and dyeing the Seine blue.^4^ In all, the pre‐Olympics statements involved a variety of popular misinformation regarding the Olympic games, including narratives on athlete suspensions, illegal drug testing, and unsubstantiated security threats.

**TABLE 1:** Experimental assignment at $T_{1}$ (pre‐Olympics) and $T_{2}$ (post‐Olympics).

Group	Outcome measure	$T_{1}$ stimuli	$T_{2}$ stimuli
A1	Accuracy (backfire)	3 true fillers 3 false claims (*Set A*)	6 true fillers 6 false claims *(Set A + Set B)*
B1	Accuracy (backfire)	3 true fillers 3 false claims (*Set B*)	6 true fillers 6 false claims *(Set A + Set B)*
A2	Consensus (illusory)	3 true fillers 3 false claims (*Set A*)	6 true fillers 6 false claims *(Set A + Set B)*
B2	Consensus (illusory)	3 true fillers 3 false claims (*Set B*)	6 true fillers 6 false claims *(Set A + Set B)*

*Note*: “Outcome measure” refers to the dependent variable applied to each group's responses; it is not an additional between‐subjects factor. All groups received identical stimuli at each wave. The between‐subjects factor is the *set* of false claims seen at $T_{1}$ (Set A vs. Set B). The within‐subjects comparison at $T_{2}$ (previously seen vs. new items) provides the primary inferential test.

**FIGURE 3:**
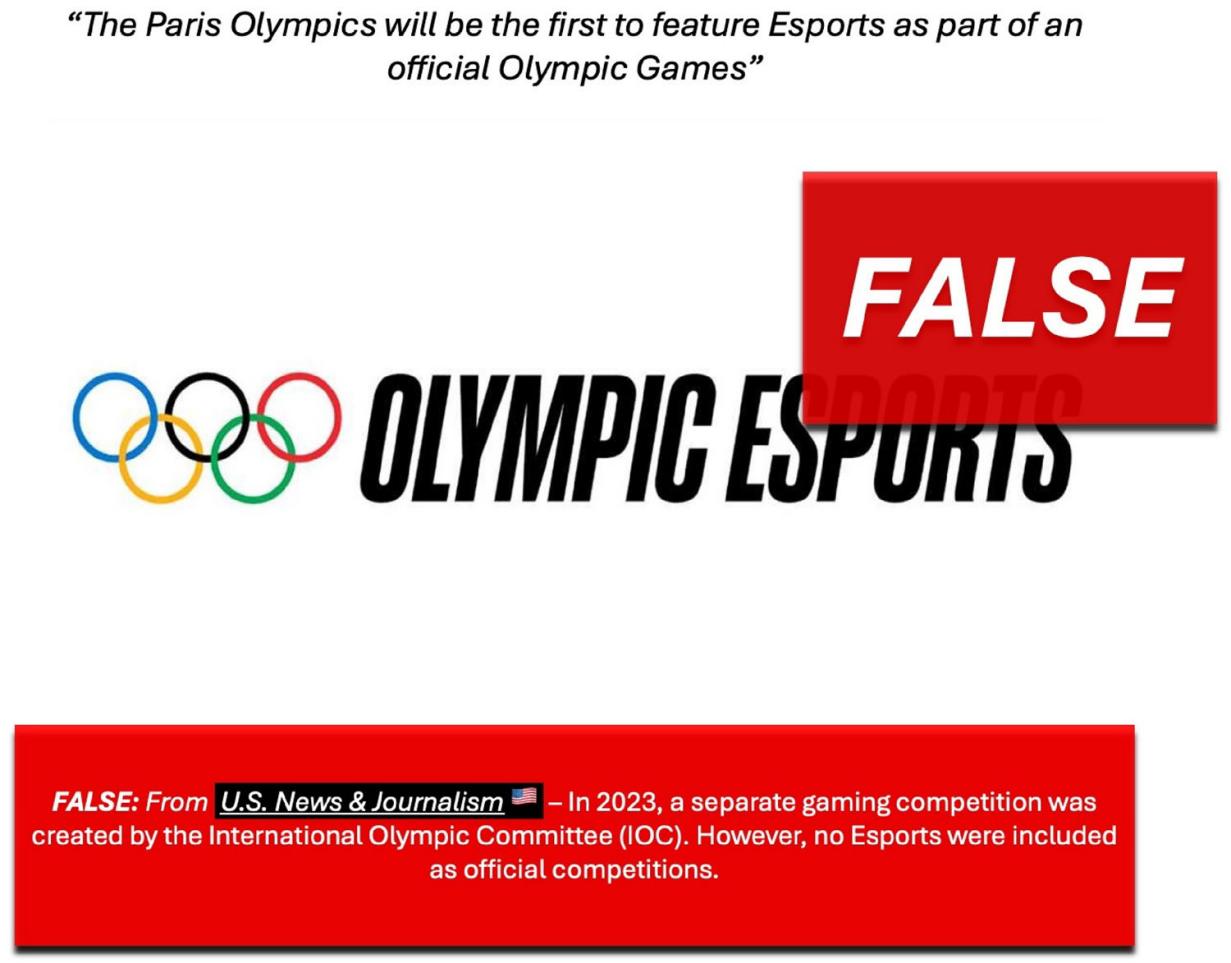
Example of misinformation claim and accompanying fact‐check.

Prior to reviewing the claims, participants were presented with the instructions for the survey experiment, which read “We will now show you a series of six posts recently made on X/Twitter. Some contain true information while others contain false information. Each post is about the upcoming Olympic Games.”^5^ For each post, participants were first asked, as a test of *H1*: “How accurate is the information in this post?” Available responses ranged on a six‐point Likert scale that ranged from *Very accurate* (1) to *Very inaccurate* (6).^6^ In addition, following this initial accuracy assessment participants were asked, as a test of *H2*, to make consensus judgments about each statement. The instructions for this question read: “If we asked 100 of your fellow citizens: [*STATEMENT*]. How many would you expect to rate the statement as containing accurate information?” Respondents were asked to report their estimation on a sliding scale ranging from 0 to 100 citizens. At the conclusion of the rating process for each claim respondents received a fact‐check detailing whether the information contained in each statement was true or false along with details on the source of the claim's veracity. As the example provided in Figure 3 illustrates, each statement was paired with an associated image, modeled on common fact‐checking procedures, and overlaid with a fact‐check following each participant's completion of the primary dependent variable questions.

As noted, unlike most prior studies on the persistence of fact‐checks, the second wave of the study took place one week after the focal event (the Olympic Games) to provide the study with greater ecological validity. In this follow‐up wave, returning participants were each presented with twelve statements. These statements included six true statements and six false statements. The six true statements were further split between the three true statements they had previously seen fact‐checked and three new statements. Similarly, the false statements were split between the three statements they had previously seen fact‐checked in the first wave and the three false statements each participant had not seen in the first wave. Participants were again prompted to answer the primary DV questions on accuracy and consensus for each statement.

### 5.3 Analytical Approach

In our primary analyses, we treat each participant as their own control and subject the data to within‐subjects paired *t*‐tests. Every respondent encounters six false claims at $T_{2}$ (post‐Olympics). This included the three false claims they had already seen and were fact‐checked on at $T_{1}$ and the three new claims they had not seen. For each participant we compute two means at $T_{2}$: the average 1–6 accuracy judgment and the average 0–100 consensus estimate, separately for the old and the new items. A two‐tailed paired *t*‐test on these within‐person differences provides the primary test of the backfire hypothesis. A negative difference on the accuracy scale would reveal that prior correction increases belief in misinformation (evidence of a “backfire effect”). Alternatively, a positive difference on the consensus scale would provide evidence of the presence of an illusory consensus, that is, that corrections lead people to over‐estimate how widely the claim is believed.

In addition to the paired *t*‐tests specified in the preanalysis plan, we report results from linear mixed‐effects models with participant random intercepts and claim random effects. This approach accounts for claim‐level heterogeneity and uses all observations rather than person‐level aggregates, providing a more efficient test while yielding consistent substantive conclusions. We further supplement these with model specifications that include set assignment (A vs. B) as an interaction term to test whether the specific composition of claims presented at $T_{1}$ moderated the observed effects. Results persist across specifications.^7^.

**FIGURE 4:**
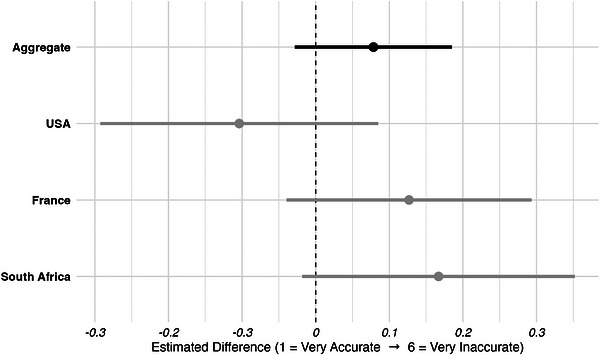
Test of the backfire effect. *Note*. Error bars represent the estimated difference with 95% CIs.

**FIGURE 5:**
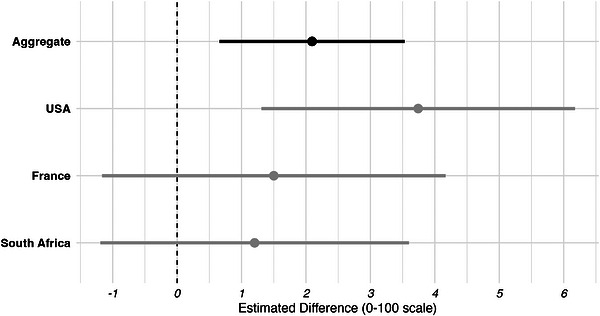
Test of the illusory consensus effect. *Note*. Error bars represent the estimated difference with 95% CIs.

## 6 Results

Using this dataset, we first assessed whether there was evidence of a backfire effect between the pre‐ and post‐Olympics survey waves.

Across the 606 respondents who completed the post‐Olympics wave, the mean accuracy rating for previously seen, fact‐checked claims (M = 3.86 on the 1–6 scale) exceeded the mean rating for new, unseen claims (M = 3.79) by 0.068 points. A paired *t*‐test shows that this difference is not statistically distinguishable from zero (t(605) = 1.18, *p* = 0.24; 95% CI = [−0.045, 0.181]; d = 0.048). Substantively, the point estimate implies a backfire of roughly one‐twelfth of a scale unit, under one‐fifth of a standard deviation. At the same time, the confidence interval comfortably spans both modest negative and modest positive effects. We therefore find no evidence that prior correction increased belief in misinformation, consistent with the majority of recent replications that fail to detect classic backfire effect (see Figure 4).^8^

To examine whether fact‐checking effects varied across specific misinformation narratives, we conducted item‐level between‐subjects tests comparing $T_{2}$ responses for participants who had versus had not seen each claim fact‐checked at $T_{1}$ (see Appendix Tables 9 and 10 in the Supporting Information for full results). These analyses reveal substantial heterogeneity across claims. Two claims (the COVID‐19 vaccine requirement (d = 0.39) and the Celsius energy drink ban (d = 0.17)) showed significant effects in the direction *opposite* to backfire, indicating that fact‐checks for these particular claims produced lasting reductions in false belief. The remaining four claims showed no significant differences. None of the six claims showed evidence of a backfire effect, though the CIA warnings (d = −0.13) and transgender athletes (d = −0.11) claims trended nonsignificantly in that direction. This heterogeneity underscores the importance of considering content‐specific factors when evaluating fact‐checking effectiveness and is further explored in the set‐level comparison below.

To evaluate whether the specific set of false claims presented at $T_{1}$ influenced the magnitude of the observed effects, we compared effect sizes between participants randomly assigned to Set A and Set B. The backfire difference score was significantly larger for Set B participants (M = 0.415) than Set A participants (M = −0.267; t(602.3) = −6.10, p < 0.001). This indicates that claims in Set B (which included the COVID‐19 vaccine and Celsius energy drink claims) showed a pattern more consistent with fact‐check persistence, while Set A claims showed no such pattern. However, when tested within the linear mixed‐effects framework that accounts for claim‐level heterogeneity through random effects, the seen × set interaction was not statistically significant (β = 0.685, t = 1.30), suggesting that the set‐level differences are attributable to variation across individual claims rather than a systematic set‐level effect.

Respondents estimated that previously seen, fact‐checked claims would be believed by 2.3% more of their compatriots than brand‐new claims introduced only at $T_{2}$ (β = 2.28, 95% CI [0.83, 3.72]; d = 0.125). The effect is statistically significant (t(605) = 3.08, *p* = 0.002). This result provides evidence that fact‐checks can produce an illusory consensus. That is, in the case of Olympic misinformation, corrections increased perceptions of societal agreement with misinformation. Substantively, however, the shift is small. On a 0–100 scale, the average estimate rose from roughly 54%–56% when aggregated across country cohorts (see Figure 5).

A parallel pattern to that observed for the backfire effect emerged at the item level for the illusory consensus effect. The Blue Seine (d = 0.15) and Esports (d = 0.14) claims showed the strongest tendencies toward illusory consensus, while the COVID‐19 vaccine claim trended in the opposite direction, though none reached conventional significance at the individual‐claim level. For the consensus effect, the aggregate Set A versus Set B comparison was also significant (t(598.4) = 4.56, p < 0.001), with Set A showing a positive consensus distortion (M = 5.54, d = 0.320) and Set B showing none (M = −1.10, d = −0.059). However, item‐level analyses suggest this set difference should be interpreted with caution as five of the six false claims showed a consistent trend toward illusory consensus (d = 0.092–0.144), with no clear distinction between Set A and Set B items on this dimension. The notable exception was the COVID‐19 vaccine claim, which exhibited the opposite pattern (d = −0.101). This single anomalous item drives the Set B aggregate to null, as the vaccine claim's negative effect offsets the positive consensus trends for the Celsius (d = 0.092) and Blue Seine (d = 0.144) claims. Within the linear mixed‐effects framework, the seen × set interaction was not statistically significant for the consensus outcome (β = −4.995, t = −0.42), reinforcing that the set‐level differences reflect individual claim variation rather than a systematic set effect.

### 6.1 Supplementary Analysis: Effects of Fact‐Checking on True Statements

The existing literature on fact‐checking has focused predominantly on the correction of false claims, with comparatively little attention to how fact‐checking verified information shapes subsequent beliefs. Pennycook et al. (2020) demonstrated that selectively labeling some headlines as false produces an “implied truth effect,” whereby unlabeled content is perceived as more accurate. Critically, Pennycook et al. (2020) found that this problem could be countered by explicitly tagging true headlines as “verified,” thereby removing the inferential ambiguity that arises when only false content is labeled. More broadly, while a growing literature has examined the durability of corrections to misinformation, often finding that effects decay within days or weeks (Guess et al. 2020; Swire‐Thompson et al. 2020), the persistence of fact‐checking effects on true statements across a multi‐week, ecologically valid design has received less attention.

To address this gap, we assessed the effects of fact‐checking on true statements using two complementary comparisons, each addressing a distinct question. The first, which compares $T_{2}$ ratings of previously confirmed true statements against participants' own $T_{1}$ ratings of the same items, tests whether fact‐checking produced a *durable shift* in beliefs over time. The second, which compares $T_{2}$ ratings of previously confirmed true statements against new true statements encountered for the first time at $T_{2}$, tests whether the benefit of prior fact‐checking is attributable to the intervention itself rather than to mere familiarity or repeated exposure, since both sets of items are rated at the same time point but only one received the fact‐check treatment.

For accuracy judgments, the pre–post comparison revealed that participants rated previously confirmed true statements as substantially more accurate at $T_{2}$ (M = 2.20 on the 1–6 scale) compared to $T_{1}$ (M = 3.13), representing a large and significant improvement (t(618) = −17.63, p < 0.001, d = −0.71). Critically, the within‐$T_{2}$ comparison yielded an even larger effect, as previously confirmed true statements were rated as significantly more accurate than new, unverified true statements (M = 3.40; t(618) = −22.74, p < 0.001, d = −0.91). The fact that the within‐$T_{2}$ comparison (which controls for general time‐of‐testing effects) produced the larger effect strengthens the inference that the improvement is attributable to the fact‐check itself rather than to repeated exposure or familiarity.

For consensus estimates, a complementary pattern emerged. Participants estimated that previously confirmed true statements would be believed by substantially more citizens at $T_{2}$ (M = 69.41) compared to $T_{1}$ (M = 59.88; t(618) = 11.51, p < 0.001, d = 0.46). At $T_{2}$, previously confirmed true statements were also estimated to have greater public acceptance than new true statements (M = 54.90; t(618) = 18.04, p < 0.001, d = 0.72). Again, the within‐$T_{2}$ comparison yielded the larger effect, reinforcing that the consensus shift reflects the influence of fact‐checking rather than mere re‐exposure.

**FIGURE 6:**
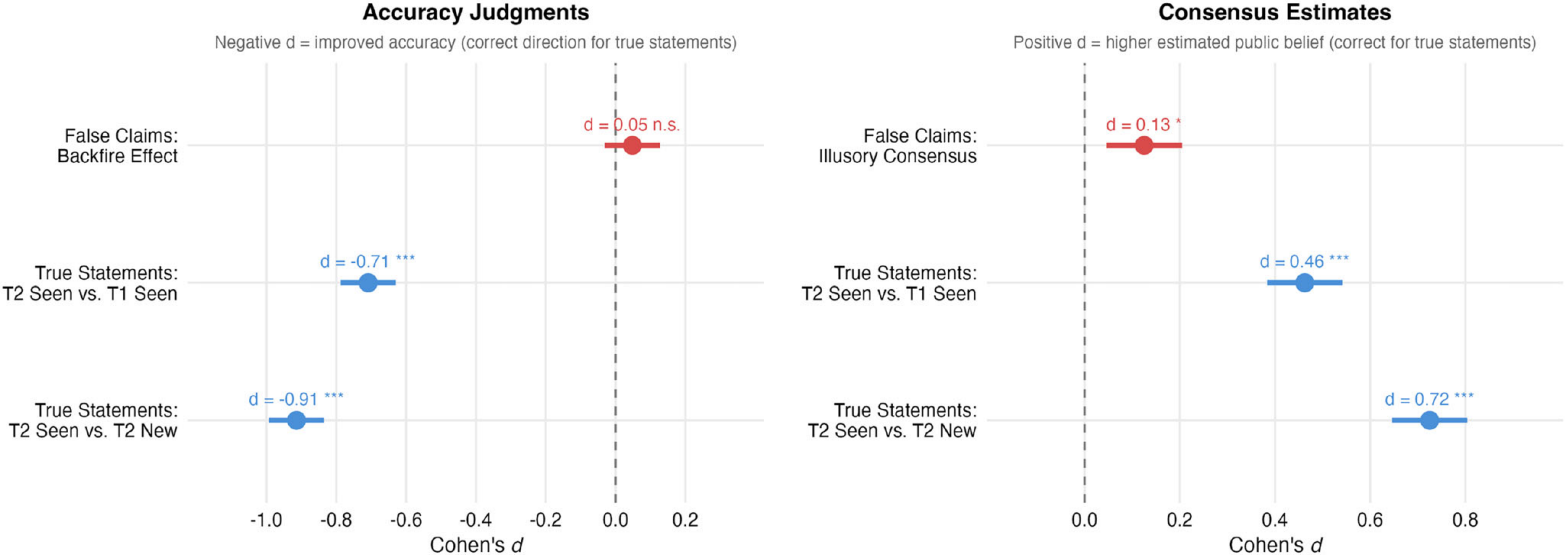
Comparison of fact‐checking effects: False claims versus true statements. Left panel: accuracy judgments (Cohen's d; negative values indicate improved accuracy). Right panel: consensus estimates (positive values indicate higher estimated public belief). Red = false claims (backfire and illusory consensus tests). Blue = true statements (fact‐check confirmation effects). Error bars represent 95% CIs.

Figure 6 visualizes the contrast between fact‐checking effects on false claims and true statements across both outcome measures. The negligible backfire effect (d = 0.05) and small illusory consensus effect (d = 0.13) for false claims stand in stark contrast to the large, durable effects observed for true statements (d = 0.71–0.91 for accuracy, d = 0.46–0.72 for consensus). These findings demonstrate that, at least in this case, the fact‐checking intervention was substantially more effective at reinforcing correct beliefs than they were at inadvertently entrenching falsehoods or distorting meta‐perceptions. This extends the work of Pennycook et al. (2020) by demonstrating that the benefits of verification labels persist across weeks in a naturalistic context. Moreover, whereas prior research has documented rapid decay of corrections to false claims (Guess et al. 2020; Swire‐Thompson et al. 2020), the large effect sizes observed here suggest that verification of true information may be considerably more durable. This asymmetry has important practical implications for the design and evaluation of fact‐checking interventions.

## 7 Discussion and Conclusion

This work aimed to extend prior research on the potential downsides of fact‐checking in the aim of ensuring that the benefits of this essential corrective practice are isolated from potential drawbacks. Prior work on these negative consequences has relied primarily on laboratory and survey studies which present decontextualized falsehoods to participants. To improve the validity of these findings, we examined two related, but separate potential challenges tied to the need for fact‐checks (and related debunking messages): the backfire effect and the illusory consensus effect. Critically, we examine these phenomenon in the context of the 2024 Olympic Games, which provided a real‐world case involving active misinformation narratives. Using a cross‐country panel design, which provides further validity and context to our results, our study provides a novel test of longstanding fears related to the backfire effect and novel evidence regarding the potential for fact‐checks to produce an “illusory consensus” among readers.

Contrary to earlier concerns in the misinformation literature, our study found no evidence to support the backfire effect. The aggregate effect was negligible (d = 0.048). While the point estimate was in the direction consistent with fact‐check persistence rather than backfire (previously corrected items were rated slightly *more* inaccurate than new items), we emphasize that this should be interpreted as an absence of evidence for backfire rather than definitive evidence that backfire cannot occur. If a backfire effect does exist in contexts like these, our data suggest it is likely to be very small. These null results are consistent with the majority of recent replications and add to the preponderance of evidence that the backfire effect is unlikely to pose a meaningful threat to standard fact‐checking practices.

On the other hand, the data also indicate the presence of a small “illusory consensus effect” in the aggregate (d = 0.125), corresponding to an increase of approximately 2.3%. Participants exposed to fact‐checks about misinformation circulating online about the Olympic Games were subsequently more likely to estimate that the wider population would accept these debunked claims as accurate. However, this aggregate effect masks heterogeneity at both the country and item levels. The consensus distortion was concentrated among US participants (d = 0.219, p = 0.003), while it did not reach statistical significance in either the French (d = 0.109, p = 0.133) or South African (d = 0.068, p = 0.303) cohorts. At the item level, five of the six false claims trended in the illusory consensus direction (d = 0.092–0.144), suggesting a small, diffuse tendency. The notable exception was the COVID‐19 vaccine claim, which exhibited the opposite pattern (d = −0.101). The divergent behavior of the vaccine claim may reflect the unique informational environment surrounding COVID‐19, where extensive public health messaging and politicized discourse may have provided participants with additional corrective signals beyond the experimental fact‐check itself.

We note that even small per‐exposure distortions to consensus perceptions could, in principle, compound across repeated encounters with fact‐checks during contentious public events, particularly given established links between inaccurate meta‐perceptions and behaviors such as self‐censorship (Lees and Cikara 2020) and support for political violence (Mernyk et al. 2022). At the same time, the modest absolute magnitude of the effect, the fact that no individual claim reached significance on its own, and the sensitivity of the aggregate to a single anomalous item counsel against interpreting it as a robust general threat to fact‐checking efficacy. Accordingly, while the illusory consensus finding warrants further investigation—particularly across different misinformation domains and cultural contexts—we caution against overstating its practical significance.

An important complement to these findings comes from our supplementary analyses of true statements (see Figure 6). Fact‐checking interventions that confirmed the accuracy of true claims produced large, durable effects on both accuracy judgments (d = 0.71–0.91) and consensus estimates (d = 0.46–0.72). Notably, for both outcome measures, the within‐$T_{2}$ comparison (previously confirmed vs. new true statements) yielded a larger effect than the pre–post comparison, indicating that the observed improvements are attributable to the fact‐check treatment itself rather than to familiarity or repeated exposure. These results extend the work of Pennycook et al. (2020), who demonstrated that “verified” labels can counteract the implied truth effect within a single session, by showing that verification effects persist across weeks in a naturalistic, multi‐wave context. Moreover, whereas prior research has documented rapid decay of fact‐checking effects on false claims, often within days (Guess et al. 2020), the large effect sizes observed here suggest that verification of true information may be considerably more durable. The magnitude of these effects stands in contrast to the negligible backfire effect and the small consensus distortion observed for false claims, suggesting that fact‐checking interventions are considerably more effective at reinforcing correct beliefs than they are at inadvertently entrenching falsehoods or distorting meta‐perceptions. For practitioners and platforms, this finding provides additional reassurance by highlighting how fact‐checking appears to produce its intended effects for accurate information at a scale far larger than any unintended consequences for inaccurate information.

An important limitation of this study concerns the generalizability of the Olympic misinformation context. While the Olympics provided ecological validity through real‐world misinformation narratives, many of the claims tested—for instance, whether the Seine was dyed blue or whether Esports were included in the games—may not have been deeply consequential to participants. Our item‐level analyses reveal that while five of the six false claims showed a consistent trend toward illusory consensus (d = 0.092–0.144), the COVID‐19 vaccine claim exhibited the opposite pattern (d = −0.101). This divergence likely reflects the unique informational environment surrounding COVID‐19 misinformation, where participants may have had extensive prior exposure to public health messaging and politicized discourse that provided corrective signals beyond those in our experimental manipulation. The sensitivity of the aggregate consensus result to this single claim underscores how claim‐specific factors can shape findings in ways that may not generalize. In domains where the background informational context differs (such as climate change, election integrity, or emerging health threats with little prior public messaging) both backfire and consensus effects could plausibly differ in magnitude and direction. Future research should systematically vary the domain, emotional intensity, and prior public salience of misinformation content to establish these boundary conditions.

In addition to these primary results, we also find that neither digital nor AI literacy, numeracy, science literacy, nor cognitive reflection significantly moderated the impact of fact‐checking. This lack of interaction between individual difference factors and fact‐check outcomes highlights that fact‐checking interventions may be robust across varying levels of familiarity with digital or scientific domains. However, while these measures did not moderate the *treatment effect* for false claims, bivariate correlations revealed that AI knowledge, statistical numeracy (BNT‐S), cognitive reflection (CRT), and science literacy were each significantly associated with baseline accuracy judgments and consensus estimates at both time points (see Appendix in the Supporting Information). Participants scoring higher on these measures were better at identifying false claims as inaccurate and estimated lower societal acceptance of misinformation. These associations suggest that while literacy measures do not differentially alter the effectiveness of fact‐checks for false content, they do predict individual differences in susceptibility to misinformation more broadly. A parallel analysis of these literacy measures as moderators of the true‐statement effects revealed modest moderation by statistical numeracy (BNT‐S) and science literacy, whereby participants scoring higher on these measures showed slightly larger accuracy improvements following fact‐checking of true statements (see Appendix N in the Supporting Information). Taken together, these findings contribute to a more nuanced understanding of fact‐checking and misinformation dynamics. While the lack of a backfire effect is reassuring for the ongoing efforts of fact‐checking initiatives (if not also unsurprising), the presence of an illusory consensus effect points to an underexamined area of concern.

Future research could investigate interventions designed to reduce such illusory consensus, perhaps by combining corrective information with meta‐messages aimed at dispelling misperceptions about group belief norms. Given that our analyses revealed significant content‐specific variation in both backfire and consensus effects, future work should also systematically examine how the domain and emotional intensity of misinformation narratives moderate these outcomes. The contrast between our true‐statement and false‐statement findings further suggests that research on fact‐checking effectiveness would benefit from routinely examining effects on both verified and false contents, as the mechanisms and magnitudes may differ substantially. Additionally, research into the durability of illusory consensus effects over time and among different cultural or linguistic groups would help define the boundaries of this phenomenon. By addressing these questions, scholars and practitioners alike may better tailor fact‐checking strategies to mitigate both individual‐level and societal‐level susceptibility to misinformation.

## Conflicts of Interest

The authors declare no conflicts of interest.

## Supporting information

Data S1

## Endnotes

1 Preregistration can be found online at https://aspredicted.org/58qf‐kwnb.pdf.

2 Participants were linked between surveys based on their Prolific IDs to ensure consistency across waves and to reduce the potential for duplication among participants.

3 This corresponded to a rate of around $10 per hour with some variation based on average completion rates and times by country cohort.

4 A full list of each of the selected narratives, true and false, including set assignment, is contained in Appendix Table 1 in the Supporting Information.

5 A complete version of the survey is available via the online materials in the project OSF repository.

6 These scales were translated for the survey sent to French participants.

7 All data processing and analyses were conducted using R and the scripts are available via the OSF repository.

8 In our exploratory analyses related to personal literacies, we also found null effects. These results are reported in the Appendix of the Supporting Information.
