# Supplementary material for: Assessing How Fact‐Checks Influence Accuracy and Consensus Judgments: Evidence From the Olympics
Source: Risk Anal. 2026 Jun 25;46(7):e70293. doi: 10.1111/risa.70293 (PMC13305344; doi:10.1111/risa.70293)
Supplement: Supplementary file 1 — Data S1 [file RISA-46-0-s001.pdf]

Appendix: Backfire and Consensus Effects

Appendix A

Claim Type	Claim Text	Set
TRUE - 1	**Claim:** The beds assembled for Olympians in Paris's Olympic Village were made from cardboard.<br>**Factcheck:** TRUE: The cardboard beds, first used in the 2020 Tokyo Olympics, are made from fully recycled materials to promote sustainability.	–
TRUE - 2	**Claim:** The IOC Charter prohibits athletes from undertaking any and all forms of political expression.<br>**Factcheck:** TRUE: The International Olympic Committee (IOC) has banned political expressions by athletes since the 1970 Olympic Games.	–
TRUE - 3	**Claim:** The 2024 Paris Olympics will be the first to have equal numbers of male and female athletes competing.<br>**Factcheck:** TRUE: The 2024 Summer Games will be the first to have 50% female and 50% male athletes.	–
TRUE - 4	**Claim:** The Paris Olympics saw fewer world records broken than any of the previous six Olympic Games.<br>**Factcheck:** TRUE: Just 17 world records were broken at the 2024 Olympic Games, which is fewer than all other games held in the 21st Century.	–
TRUE - 5	**Claim:** The shallow depth of the Olympic pool used to host events in Paris has been blamed for slowing swimmers.<br>**Factcheck:** TRUE: While it has been debated whether the shallow depth of the pool has actually slowed swimmers, it has been pointed to as a factor potentially linked to slower than usual times for certain swimming events.	–
TRUE - 6	**Claim:** More than thirty athletes competed in the Olympics as Individual Neutral Athletes (AIN) not affiliated with specific countries.<br>**Factcheck:** TRUE: Thirty-two athletes from Belarus and Russia competed in the games but were barred from representing their countries.	–
FALSE - 1	**Claim:** Ahead of the Olympic Games in Paris, the US Central Intelligence Agency released a warning recommending that Americans avoid the metro system due to an elevated threat of terrorism.<br>**Factcheck:** FALSE: No safety warnings were issued by the CIA to Americans traveling to Paris for the Summer Olympic Games.	A
FALSE - 2	**Claim:** The Paris Olympics will be the first to feature Esports as a part of an official Olympic Games.<br>**Factcheck:** FALSE: In 2023, a separate gaming competition was created by the International Olympic Committee (IOC). However, no Esports were included as official competitions.	A
FALSE - 3	**Claim:** Several female swimmers have threatened to withdraw from the Olympics if transgender athletes are permitted to compete in events.<br>**Factcheck:** FALSE: The participation of athletes is set by IOC standards and has not produced backlash from athletes threatening to withdraw.	A
FALSE - 4	**Claim:** The NCAA and Olympics have banned athletes from consuming Celsius energy drinks due to its use of banned ingredients, including carnitine and taurine.<br>**Factcheck:** FALSE: Celsius has not been banned by the IOC or the NCAA. Carnitine and taurine are not banned substances.	B
FALSE - 5	**Claim:** The International Olympic Committee (IOC) will require athletes to be vaccinated for COVID-19 to reside with other athletes in the Paris Olympic Village.<br>**Factcheck:** FALSE: Athletes will not be required to be vaccinated for COVID-19 in order to compete or reside at the Olympic Village.	B
FALSE - 6	**Claim:** A Paris City Committee has begun to dye the Seine black ahead of Olympic Swimming events set to take place later this month.<br>**Factcheck:** FALSE: While resources have been spent by the city to improve the Seine's water quality, this has not included efforts to dye the water black.	B

TABLE 1: Claim Stimuli Type, Set Assignment, and Text

Note. "–" indicates true claims presented to all participants.

Appendix B

TABLE 2: Paired T-Test: Backfire Effect

	**Estimate**	**95% CI**
Mean Difference	0.068	[−0.045, 0.181]
t (df = 605)	1.184	—
p-value	0.24	—
Cohen's d	0.048	—

Table 2 presents the results of a paired t-test looking at the difference in factchecked and non-factchecked content in the context of the *backfire effect*. Specifically, details the difference in personal belief in false content which participants viewed as factchecked misinformation in the pre-Olympics period against new false narratives.

Appendix C

TABLE 3: Paired T-Test: Illusory Consensus Effect

	**Estimate**	**95% CI**
Mean Difference	2.275	[0.782, 3.725]
t (df = 605)	3.082	—
p-value	0.002	—
Cohen's d	0.125	—

Table 3 presents the results of a paired t-test looking at the difference in factchecked and non-factchecked content in the context of the *illusory consensus effect*. Specifically, details the difference expected belief among the wider population in false content which participants viewed as factchecked misinformation in the pre-Olympics period against new false narratives.

Appendix D

TABLE 4: Literacy Measures as Moderators of the Backfire Effect

Literacy Measure	Estimate	SE	95% CI Low	95% CI High	*t*
Digital Literacy	0.083	0.103	-0.120	0.285	0.804
AI Knowledge	-0.264	0.297	-0.848	0.320	-0.888
CRT Score 7	0.043	0.028	-0.012	0.099	1.538
CRT Score 3	0.059	0.055	-0.050	0.167	1.061
BNT-S Score	0.003	0.029	-0.055	0.061	0.092
Science Literacy	0.087	0.052	-0.014	0.188	1.684

Table 4 presents results from linear regression models testing whether literacy measures predict the backfire effect (calculated as the difference in accuracy between seen and unseen items at post-test). None of the literacy measures significantly predicted the backfire effect. Science Literacy showed the strongest relationship (β = 0.087, t = 1.684, p = .093), with a marginally significant positive coefficient suggesting that higher science literacy may be associated with a slightly larger backfire effect. However, this effect did not reach conventional levels of statistical significance. CRT Score 7 also showed a trending positive relationship (β = 0.043, t = 1.538, p = .125). All other literacy measures showed weak and non-significant associations with the backfire effect.

Appendix E

TABLE 5: Paired T-Test: Backfire Effect by Country

**Country**	**Mean diff.**	**95% CI Low**	**95% CI High**	***t***	**df**	***p***	***d***
France	0.147	-0.047	0.341	1.496	194	0.136	0.107
South Africa	0.165	-0.032	0.361	1.650	230	0.100	0.109
USA	-0.099	-0.288	0.090	-1.031	191	0.304	-0.074

Table 5 presents the results of a paired *t*-test looking at the difference in factchecked and non-factchecked content in the context of the *backfire effect* across the three country cohorts included in the study. Specifically, details the difference in personal belief in false content which participants viewed as factchecked misinformation in the pre-Olympics period against new false narratives for participants from France, the United States, and South Africa. None of the selected countries show evidence that there was a backfire effect.

TABLE 6: Paired T-Test: Illusory Consensus Effect by Country

**Country**	**Mean diff.**	**95% CI Low**	**95% CI High**	***t***	**df**	***p***	***d***
France	1.911	-0.721	4.544	1.432	194	0.154	0.109
South Africa	1.234	-1.176	3.645	1.009	230	0.314	0.068
USA	3.637	1.201	6.073	2.945	191	0.004	0.219

Table 6 presents the results of a paired *t*-test looking at the difference in factchecked and non-factchecked content in the context of the *false-consensus effect* across the three country cohorts included in the study. Specifically, details the difference expected belief among the wider population in false content which participants viewed as factchecked misinformation in the pre-Olympics period against new false narratives for participants from France, the United States, and South Africa.

Appendix F

TABLE 7: Demographic Characteristics by Country

**Country**	**Age**			**Duration (Seconds)**			**Gender (%)**		
	**Mean**	**Min**	**Max**	**Mean**	**Min**	**Max**	**Female**	**Male**	**Other**
France	32.54	21	70	1171	402	8671	46.07	50.26	2.09
South Africa	29.98	21	70	1507	384	3431	47.37	51.75	0.00
USA	46.78	21	70	1039	369	4061	49.20	46.52	3.74

Table 7 presents demographic characteristics of participants by country. The sample shows notable variation in age across countries, with USA participants being substantially older on average (M = 46.78 years) compared to participants from South Africa (M = 29.98) and France (M = 32.54). Survey completion duration also varied by country, with South African participants taking the longest on average (M = 1507 seconds) and USA participants completing most quickly (M = 1039 seconds). Gender distribution was relatively balanced across all three countries, with female participants comprising between 46-49% of each country sample.

TABLE 8: Demographic Characteristics by Treatment Group

**Treatment**	**Age**			**Duration (Seconds)**			**Gender (%)**		
	**Mean**	**Min**	**Max**	**Mean**	**Min**	**Max**	**Female**	**Male**	**Other**
Backfire	36.36	21	70	1253	369	8671	48.38	49.03	1.30
Consensus	35.59	21	70	1259	384	3206	46.80	50.17	2.36

Table 8 presents demographic characteristics by treatment group assignment. The backfire and consensus groups showed similar demographic profiles, with comparable mean ages (36.36 vs 35.59 years), survey completion times (1253 vs 1259 seconds), and gender distributions (approximately 48-49% female in both groups). This balance across treatment conditions suggests successful randomization and minimal systematic differences between the groups that could confound treatment effects.

Appendix G

TABLE 9: Independent Samples T-Test: Backfire Effect by Question

Question	Mean diff.	95% CI Low	95% CI High	*t*	df	*p*	*d*
COVID-19 Vaccines	0.722	0.429	1.015	4.844	608	0.000	0.39
Celcius Energy	0.301	0.020	0.582	2.102	610	0.036	0.17
CIA Warnings	-0.229	-0.503	0.044	-1.647	607	0.100	-0.13
Trans Athletes	-0.187	-0.458	0.084	-1.357	615	0.175	-0.11
black Seine	-0.106	-0.393	0.180	-0.729	600	0.466	-0.06
Esports Games	-0.013	-0.313	0.286	-0.086	616	0.931	-0.01

Table 9 presents independent samples t-tests examining the backfire effect separately for each of the six false claims. Unlike the within-subjects comparisons in the primary analyses, these tests compare responses at Post between participants who were randomly assigned to see each specific claim fact-checked at Pre versus those who were not. This between-subjects approach at the item level complements the main within-person analysis by revealing which specific misinformation narratives may be driving the overall pattern of results. The analysis reveals substantial heterogeneity in treatment effects across different misinformation narratives. Two claims showed significant effects in the direction opposite to backfire: the COVID-19 vaccine requirement claim (mean difference = 0.722, $p < 0.001$) and the Celsius energy drink ban claim (mean difference = 0.301, $p = 0.036$), indicating that fact-checks for these claims persisted in reducing belief. The remaining four claims showed no significant differences between previously fact-checked and new items. Notably, none of the claims showed evidence of a backfire effect (negative mean differences were non-significant), though the CIA warnings and transgender athletes claims trended in that direction.

TABLE 10: Independent Samples T-Test: Illusory Consensus Effect by Question

Question	Mean diff.	95% CI Low	95% CI High	*t*	df	*p*	*d*
COVID-19 Vaccines	-2.665	-6.837	1.507	-1.255	609	0.210	-0.101
Celcius Energy	2.497	-1.530	6.525	1.218	614	0.224	0.092
CIA Warnings	2.860	-1.025	6.746	1.446	617	0.149	0.116
Trans Athletes	2.197	-1.559	5.954	1.149	616	0.251	0.098
black Seine	3.979	-0.376	8.333	1.794	610	0.073	0.144
Esports Games	3.841	-0.573	8.255	1.709	616	0.088	0.137

Table 10 presents independent samples t-tests examining the illusory consensus effect separately for each false claim. As with Table 9, these tests compare responses between participants who did versus did not see each specific claim at Pre, providing a between-subjects perspective that complements the within-person comparisons in the main analysis. The item-level analysis reveals considerable variation across claims, though none reached conventional statistical significance. The black Seine dyeing claim (mean difference = 3.979, $p = 0.073$) and Esports claim (mean difference = 3.841, $p = 0.088$) showed the strongest tendencies toward illusory consensus effects,

while the COVID-19 vaccine claim showed a non-significant trend in the opposite direction. This heterogeneity suggests that the illusory consensus effect documented in the aggregate analysis may be driven more strongly by certain types of misinformation narratives than others.

Appendix H

TABLE 11: Paired T-Test: Backfire Effect - Related Narratives

	**Estimate**	**95% CI**
Mean Difference	-0.003	[−0.116, 0.110]
t (df = 605)	-0.049	—
p-value	0.961	—

Table 11 presents the results of a paired t-test looking at the difference in content related to fact-checked and non-fact-checked content. Specifically, details the difference in personal belief in false content which participants viewed content *related* to fact-checked misinformation in the pre-Olympics period against new false narratives.

TABLE 12: Paired T-Test: Illusory Consensus Effect - Related Narratives

	**Estimate**	**95% CI**
Mean Difference	0.931	[−0.555, 2.418]
t (df = 592)	1.230	—
p-value	0.219	—

Table 12 presents the results of a paired t-test looking at the difference in content related to fact-checked and non-fact-checked content. Specifically, details the difference expected belief among the wider population among content *related* to false content which participants viewed as factchecked misinformation in the pre-Olympics period against new false narratives.

Appendix I

TABLE 13: Linear Mixed-Effects Model: Backfire Effect Test

Predictor	Estimate	SE	95% CI Low	95% CI High	t	p
Previously Fact-Checked	0.078	0.054	-0.028	0.185	1.441	0.150

Table 13 presents results from a linear mixed-effects model testing for the presence of a backfire effect. The model includes fixed effects for each claim and random intercepts for participants (Participant SD = 0.729, Residual SD = 1.650). Unlike the paired t-test, which aggregates responses to person-level means, this approach uses all individual claim-level responses and accounts for both participant-level clustering and systematic differences across specific claims. The model estimates that previously fact-checked false claims were rated 0.078 points higher on the 1–6 inaccuracy scale compared to new false claims, though this difference was not statistically significant ($p = 0.150$). The positive coefficient indicates that, if anything, fact-checks persisted rather than backfired, consistent with findings from the paired t-test.

TABLE 14: Linear Mixed-Effects Model: Illusory Consensus Test

Predictor	Estimate	SE	95% CI Low	95% CI High	t	p
Previously Fact-Checked	2.165	0.744	0.708	3.623	2.912	0.004

Table 14 presents results from a linear mixed-effects model testing for the presence of an illusory consensus effect. The model includes fixed effects for each claim and random intercepts for participants (Participant SD = 13.038, Residual SD = 22.495). Unlike the paired t-test, which aggregates responses to person-level means, this approach uses all individual claim-level responses and accounts for both participant-level clustering and systematic differences across specific claims. The model estimates that previously fact-checked false claims were perceived as being believed by 2.165 more citizens (out of 100) compared to new false claims, and this difference was statistically significant ($p = 0.004$). The positive coefficient provides evidence of an illusory consensus effect, wherein exposure to fact-checks leads participants to overestimate how widely misinformation is believed in broader society.

Appendix J: Set A vs. Set B Comparison

TABLE 15: Paired T-Test: Backfire Effect by Set Assignment

Set	*n*	Mean diff.	95% CI Low	95% CI High	*t*	*p*	*d*
Set A	308	-0.267	-0.412	-0.122	-3.62	<.001	-0.206
Set B	298	0.415	0.256	0.573	5.15	<.001	0.298
A vs. B					-6.10	<.001	

TABLE 16: Paired T-Test: Illusory Consensus Effect by Set Assignment

Set	*n*	Mean diff.	95% CI Low	95% CI High	*t*	*p*	*d*
Set A	308	5.54	3.22	7.86	4.69	<.001	0.320
Set B	298	-1.10	-3.21	1.01	-1.03	.305	-0.059
A vs. B					4.56	<.001	

Tables 15–16 present paired t-tests for the backfire and illusory consensus effects separately by set assignment. Set A participants (CIA Warning, Esports, Transgender Athletes) showed a pattern consistent with fact-check persistence for accuracy but a significant illusory consensus effect. Set B participants (Celsius, COVID-19 Vaccine, black Seine) showed significant fact-check persistence for accuracy but no consensus distortion. The divergent consensus results for Set B are driven by the anomalous behavior of the COVID-19 vaccine claim (see Appendix G, Table 10).

Appendix K: Linear Mixed-Effects Models with Set Interaction

TABLE 17: LMM: Backfire Effect with Set × Seen Interaction

Predictor	Estimate	SE	95% CI Low	95% CI High	*t*	*p*
Seen (Fact-Checked)	0.078	0.054	-0.028	0.185	1.44	0.150
Set B						
Seen × Set B	0.685	0.527	-0.348	1.718	1.30	0.194

TABLE 18: LMM: Consensus Effect with Set × Seen Interaction

Predictor	Estimate	SE	95% CI Low	95% CI High	*t*	*p*
Seen (Fact-Checked)	2.165	0.744	0.708	3.623	2.91	0.004
Set B						
Seen × Set B	-4.995	11.850	-28.240	18.250	-0.42	0.673

Tables 17–18 present linear mixed-effects models that include the seen × set assignment interaction. Models include participant random intercepts and claim random effects. The seen × set interaction was not statistically significant for either the backfire (t = 1.30) or consensus (t = -0.42) outcome, indicating that set-level differences observed in the aggregate comparisons are attributable to variation across individual claims rather than a systematic difference between the two sets.

Appendix L: True Statement Analyses

TABLE 19: Paired T-Test: True Statement Accuracy Judgments

**Comparison**	**Mean diff.**	**95% CI Low**	**95% CI High**	***t***	**df**	***d***
Pre vs. Post (T_1 to T_2)	-0.93	-1.03	-0.82	-17.63	618	-0.71
Seen vs. New at T_2	-1.20	-1.31	-1.10	-22.74	618	-0.91

Table 19 presents paired t-tests for accuracy judgments of true statements. Lower values indicate greater perceived accuracy (1 = Very Accurate, 6 = Very Inaccurate). Previously confirmed true statements were rated significantly more accurate at T_2 than at T_1 (d = -0.71, $p < .001$), and were rated more accurate than new, unverified true statements introduced at T_2 (d = -0.91, $p < .001$).

TABLE 20: Paired T-Test: True Statement Consensus Estimates

**Comparison**	**Mean diff.**	**95% CI Low**	**95% CI High**	***t***	**df**	***d***
Pre vs. Post (T_1 to T_2)	9.53	7.91	11.16	11.51	618	0.46
Seen vs. New at T_2	14.51	12.93	16.09	18.04	618	0.72

Table 20 presents paired t-tests for consensus estimates of true statements. Higher values indicate that more fellow citizens were expected to rate the statement as accurate (0–100 scale). Previously confirmed true statements showed significantly higher estimated consensus at T_2 than at T_1 (d = 0.46, $p < .001$), and higher consensus than new true statements at T_2 (d = 0.72, $p < .001$).

Appendix M: Individual Difference Correlations

TABLE 21: Bivariate Correlations: Individual Difference Measures and Outcome Variables

**Measure**	**Accuracy** T_1	**Accuracy** T_2	**Consensus** T_1	**Consensus** T_2
Digital Literacy	0.045	0.021	-0.033	0.013
AI Knowledge	0.111***	0.132**	-0.073**	-0.193***
BNT-S	0.174***	0.232***	-0.165***	-0.197***
CRT-7	0.142***	0.295***	-0.138***	-0.156***
Science Literacy	0.209***	0.268***	-0.179***	-0.208***

Note. Higher accuracy scores indicate greater perceived inaccuracy (correct direction for false claims). Negative consensus correlations indicate lower estimated societal acceptance of misinformation (correct direction). * $p < .05$, ** $p < .01$, *** $p < .001$.

Table 21 presents bivariate Pearson correlations between each individual difference measure and accuracy/consensus judgments at both time points. AI knowledge, statistical numeracy (BNT-S), cognitive reflection (CRT-7), and science literacy were each significantly correlated with accuracy and consensus at both time points, indicating that higher scores on these measures predicted better identification of false claims as inaccurate and lower estimated societal acceptance of misinformation. Digital literacy showed weak, non-significant associations. While these measures did not moderate the treatment effect (i.e., the difference between seen and unseen items), they do appear to predict individual differences in susceptibility to misinformation more broadly.

Appendix N: Individual Differences and True Statement Effects

TABLE 22: Moderation of True Statement Treatment Effects by Individual Difference Measures

**Measure**	**Acc Pre–Post**	**Acc Seen–New**	**Cons Pre–Post**	**Cons Seen–New**
Digital Literacy	−0.01	−0.06	−0.03	0.03
AI Knowledge	−0.07	−0.08	0.06	0.08*
CRT-7	−0.02	−0.08*	0.11**	0.09*
BNT-S	−0.10**	−0.10**	0.08	0.09*
Science Literacy	−0.14**	−0.13**	0.06	0.05

Note. Values are Pearson correlations (r) between each literacy measure and the within-person treatment effect (difference score) for true statements. Accuracy differences: negative r indicates that higher literacy is associated with a larger accuracy improvement from fact-checking. Consensus differences: positive r indicates that higher literacy is associated with a larger increase in estimated public belief. * $p < .05$, ** $p < .01$, *** $p < .001$.

Table 22 extends the individual difference analyses to the true statement effects. Digital Literacy shows no significant moderation of any true statement treatment effect. Statistical numeracy (BNT-S) and science literacy emerge as modest moderators of accuracy improvements, with higher scores associated with slightly greater pre-post gains (BNT-S: $r = -0.10$, $p = .010$; science literacy: $r = -0.14$, $p = .001$) and slightly larger seen-versus-new differences at T_2. CRT-7 and BNT-S additionally predict slightly larger consensus shifts for previously confirmed true statements. AI knowledge shows a similar but weaker trend. However, these effects are small (r generally ≈ 0.10), and given the number of tests conducted, should be interpreted cautiously.
